# Is Lymphedema Cure a Clinical Reality?

**DOI:** 10.1055/a-2130-2113

**Published:** 2023-11-30

**Authors:** Wei F. Chen, Ying C. Ku, Takumi Yamamoto, Sonia K. Pandey

**Affiliations:** 1 Department of Plastic and Reconstructive Surgery, Cleveland Clinic Foundation, Center for Lymphedema Research and Reconstruction, Cleveland, Ohio; 2 Department of Plastic Surgery, National Center for Global Health and Medicine in Japan, Tokyo, Japan

Lymphedema surgery and the science of lymphology rapidly advanced in the past decades. Today, procedures such as supermicrosurigcal lymphaticovenular anastomosis (LVA), vascularized lymph vessel transfer (VLVT), and vascularized lymph node transfer (VLNT) are performed worldwide and are no longer considered esoteric. Despite the increased popularity of these procedures, the debate on these procedures' efficacies continues. More specifically, is lymphedema surgically curable? Here, we offer our experience and viewpoint.

In our experience, with cure defined as a patient being free of all lymphedema-related symptoms without the need for further treatments including the use of compression garments, lymphedema cure is most definitely a clinical reality. Although achievable only in a small fraction of our patients, we have seen all three procedures (LVA, VLVT, and VLNT) capable of effecting cure since 2011. 1 2 3 4 The feasibility of cure seems to correlate with the fluid-predominant state of lymphedema or stages of the pathology in which edema was fully reversible. No cure was ever observed in patients with solid-predominant disease or when the affected limbs were prominently affected by lymphedema-induced lipodystrophy. These physiologic procedures seemed not capable of reversing the pathologic lipodystrophy once developed. Interestingly, complete disease reversal was possible even in patients with advanced lymphatic injury, or those showing “diffuse” pattern on indocyanine green lymphography, provided that their conditions remained fluid predominant.

Regardless of the physiologic procedure performed, all of those who achieved cure followed a consistent time course of prompt, notable symptomatic improvement within 2 weeks from surgery. All then experienced progressive amelioration of pain, paresthesia, heaviness, clumsiness, rigidity, severity and frequency of spontaneous infection, exercise intolerance, and other related symptoms in the following 6 postoperative months. All cases of cure were documented between 6 to 18 months following surgery. None achieved cure if they were still dependent on a compression garment on the 18th month. Frequently, those who achieved cure had such insight – they knew they were ready to stop wearing compression garments and would stop doing so themselves before being given permission by us at subsequent clinic follow-up. Although remarkable improvements were consistently seen in those who underwent hybrid reconstruction (debulking procedure in combination with a physiologic procedure), fewer cure cases were observed in this patient group relative to those with fluid-predominant disease who underwent physiologic procedure only.

In our opinion, we should not be complacent with only ameliorating lymphedema. We need to aim to cure. We should review our collective experience and investigate patient/technical characteristics that are associated with cure. With these known, we hope to be able to achieve cure with increased confidence and frequency. For now, with this commentary, we hope to convey to the lymphedema surgery community that cure is a clinical reality, and it is time that we set our therapeutic bar higher.
